# A Comparative Assessment of OECD Countries’ Health Performance Through an Integrated Objective MCDM Framework

**DOI:** 10.3390/healthcare14081050

**Published:** 2026-04-15

**Authors:** Neylan Kaya, Aslıhan Ersoy Bozcuk, Güler Ferhan Ünal Uyar, Eylül Türkay, Mehtap Türkay

**Affiliations:** 1 Department of Business Administration, Faculty of Economics and Administrative Sciences, Akdeniz University, Antalya 07058, Türkiye; neylankaya@akdeniz.edu.tr (N.K.); aebozcuk@akdeniz.edu.tr (A.E.B.); guleruyar@akdeniz.edu.tr (G.F.Ü.U.); 2 Barts Cancer Institute, Queen Mary University of London, London EC1M 6BQ, UK; bt22671@qmul.ac.uk; 3 Department of Public Health, Faculty of Medicine, Akdeniz University, Antalya 07058, Türkiye

**Keywords:** health system performance, OECD countries, MCDM

## Abstract

**Highlights:**

**What are the main findings?**

- An integrated objective MCDM framework (CRITIC–MAIRCA–MARCOS) reveals substantial health system performance differences among OECD countries.
- Switzerland, Sweden, Denmark, and Japan consistently rank at the top, while Mexico and Türkiye remain at the bottom, with strong agreement between MAIRCA and MARCOS results (Spearman’s ρ = 0.959).

**What are the implications of the main findings?**

- Health spending per capita and life expectancy emerge as the most influential indicators, highlighting the importance of efficient resource allocation alongside outcome-based measures.
- The strong consistency across ranking methods demonstrates that combining objective weighting with multiple MCDM techniques provides a robust and policy-relevant framework for cross-country health system evaluation.

**Abstract:**

**Background/Objectives**: The comparative evaluation of health system performance is becoming increasingly critical for policy makers in the context of rising health expenditures, demographic ageing, and the deepening of health inequalities between countries. In the existing literature, a substantial proportion of studies addressing health performance either examine causal relationships based on single health outcomes or rely on a single multi criteria decision making (MCDM) method based on equal or subjective weighting. This situation may lead to limitations in terms of method sensitivity and the reliability of the resulting rankings. This study addresses an important gap in the literature by directly tackling method sensitivity through the integrated use of objective weighting and multiple ranking methods. The aim of this study is to evaluate the health system performance of OECD countries within an integrated MCDM framework based on objective weighting. **Methods**: The analysis covers 27 OECD countries and is based on key indicators representing health performance, such as life expectancy, avoidable mortality, infant mortality rate, and maternal mortality rate. Criterion weights are determined objectively using the CRITIC method, and country performance rankings are obtained using the MAIRCA and MARCOS methods. **Results**: The findings indicate that there are substantial differences in health system performance among OECD countries. The high level of consistency between the results obtained from different ranking methods supports the methodological robustness of the findings. **Conclusions**: In this respect, the study contributes to the literature on health system performance evaluation at both methodological and applied levels and provides policy makers with a more reliable framework for comparative analysis.

## 1. Introduction

The performance of health systems has become an increasingly important issue for policy makers and researchers in parallel with rising health expenditures, demographic ageing, and the deepening of health inequalities between countries. Evaluating the extent to which health systems can effectively transform available resources into health outcomes is critical not only for improving health results but also for the development of sustainable health policies.

In this study, the concept of “health system performance” is approached primarily from the perspective of healthcare outcomes and the effective use of health-related resources. The term “health system” may encompass broader elements such as social care, long-term care services, and public health interventions. According to the World Health Organization, a health system includes all organizations, institutions, and resources that are devoted to producing health actions aimed at improving health outcomes [1]. However, due to data comparability constraints across OECD countries, the present analysis focuses mainly on measurable healthcare outcomes and expenditure-related indicators. In this respect, the evaluation mainly reflects the effectiveness and efficiency dimensions of health systems and does not attempt to capture their full institutional and social complexity. This clarification is important to avoid overgeneralization and to ensure that the scope of the analysis remains consistent with the selected indicators. In this study, the term “health system” refers primarily to the healthcare delivery and financing structures that influence measurable health outcomes and system efficiency indicators. While broader health systems may include social care components, this analysis focuses on healthcare-related outcomes and expenditures due to data comparability constraints across OECD countries.

In addition to the general definition of health systems, international organizations such as the World Health Organization [1] and the OECD have developed performance measurement frameworks that emphasize multiple dimensions of health systems [2]. These frameworks commonly distinguish between effectiveness, efficiency, accessibility, and equity as key components of system performance. In particular, the OECD performance approach highlights that health system assessment should consider not only expenditure levels but also health outcomes and the efficient use of resources. In line with these perspectives, the present study focuses primarily on measurable outcome and resource-based indicators in order to ensure cross-country comparability within the OECD context.

In addition, OECD reports consistently document substantial cross-country differences in health outcomes and system efficiency, emphasizing that higher spending does not necessarily translate into better health performance [3,4].

Previous studies have documented strong associations between economic development and key health indicators such as life expectancy and mortality, while also highlighting substantial cross-country differences in health outcomes even among countries with similar income levels [5,6,7]. More recent comparative analyses further emphasize that health expenditure alone is insufficient to explain performance differences across OECD countries, pointing to the importance of institutional structure and system efficiency [8,9].

Although cross country comparisons of health performance have long been present in the literature, early studies predominantly focused on the relationship between economic development and health outcomes. These studies demonstrated a strong association between income per capita and indicators such as life expectancy and mortality. Over time, however, the observation of substantial differences in health outcomes even among countries with similar income levels has shown that health performance cannot be explained solely by economic growth. Factors such as institutional structure, public health policies, education level, technological advancements, and income distribution are increasingly recognized as playing a decisive role in shaping health outcomes. Indeed, comparative analyses have revealed that higher health expenditures do not always correspond to better health outcomes and that the organization and efficiency of the health system are decisive determinants of health performance. Nevertheless, a large proportion of studies in the literature addressing health performance remain limited to regression-based analyses examining the socioeconomic determinants of specific health indicators. Studies that evaluate countries’ health systems comparatively within a holistic performance measurement framework are relatively scarce. Although many existing comparative studies consider multiple indicators, they often fail to present overall health system performance through a single comprehensive measure or ranking.

In recent years, multi-criteria decision making (MCDM) methods have emerged as powerful alternative analytical tools for evaluating health system performance. These methods allow multiple indicators to be considered simultaneously and enable the comparative assessment of countries’ relative performance. However, studies employing MCDM methods remain limited in number, and a large proportion of these studies determine criterion weights using equal or subjective approaches and rely on a single ranking method. This creates certain limitations in terms of the reliability and comparability of the results. Recent empirical applications of MCDM in healthcare contexts have evaluated hospital performance, health resource allocation, and national health system comparisons using methods such as TOPSIS, VIKOR, MULTIMOORA, and DEA-based hybrids [2,10,11,12]. In addition, recent comprehensive reviews document the expanding role of multi-criteria decision analysis (MCDA) in supporting healthcare decision making and policy evaluation [13]. These studies demonstrate the analytical flexibility of MCDM frameworks; however, many remain limited either to single-country analyses or to single ranking techniques, limiting cross-country methodological comparability and robustness.

Despite the growing use of multi-criteria decision-making (MCDM) approaches in health system performance evaluation, important methodological gaps remain. First, many existing studies rely on equal or subjectively assigned criterion weights, implicitly assuming identical importance across heterogeneous health indicators. Such assumptions may distort comparative assessments, particularly when indicators differ substantially in variance and informational content. Second, most studies employ a single ranking technique, making country rankings highly sensitive to methodological choice and limiting robustness verification. Third, relatively few studies integrate objective weighting procedures with multiple complementary ranking methods within a unified analytical framework at the OECD level. Addressing these limitations requires the simultaneous use of information-based objective weighting and multiple ranking approaches to enhance methodological transparency, robustness, and policy relevance.

In this respect, the study contributes to the literature by addressing methodological limitations observed in prior cross-country health system evaluations. By integrating objective weighting with dual ranking techniques within a unified OECD-level framework, it enhances methodological transparency, reduces subjectivity in criterion weighting, and strengthens robustness verification in comparative health system analysis. Therefore, the study provides not only an empirical assessment of OECD health performance but also a replicable analytical framework for future cross-country health system research. Beyond its methodological contribution, this study also seeks to position its findings within the broader context of health system performance assessment and policy relevance. Comparative ranking frameworks are increasingly used by international organizations and policy makers to benchmark health system outcomes and to identify areas requiring structural improvement. However, the usefulness of such rankings depends critically on the transparency of weighting procedures and the robustness of the ranking methods employed.

In this context, the integrated use of objective weighting and multiple ranking techniques in this study provides a more balanced and interpretable evaluation framework. In practice, cross-country health system rankings are widely used by international organizations such as the OECD and the World Health Organization to support benchmarking, policy evaluation, and the identification of best practices. In this context, improving the methodological transparency and robustness of ranking frameworks is essential for ensuring their usefulness in evidence-based health policy making.

The primary aim of this study is to comparatively evaluate the health system performance of OECD countries using an integrated objective multi-criteria decision-making (MCDM) framework. Unlike prior studies that rely on single weighting schemes or individual ranking methods, this study integrates objective weighting with multiple MCDM techniques to enhance the robustness and reliability of cross-country health system evaluations.

Specifically, the study pursues three sub-aims: (i) to determine objective weights of selected health system indicators using the CRITIC method; (ii) to rank OECD countries using two complementary MCDM approaches (MAIRCA and MARCOS); and (iii) to assess the consistency and robustness of ranking results across different computational techniques. By addressing these aims, the study seeks not only to compare country performance levels but also to contribute methodologically to the reliability of cross-country health system evaluations.

## 2. Literature Review

A growing body of literature has focused on the development and application of composite indices and multi-criteria decision-making (MCDM) techniques for cross-country health system evaluation [14]. These approaches aim to provide systematic ranking frameworks that enable comparative performance assessment across countries. In this context, health system rankings have become increasingly relevant for benchmarking purposes, supporting policy analysis, and identifying structural differences in health outcomes and resource allocation efficiency. However, despite their widespread use, existing ranking frameworks often face methodological limitations, particularly regarding the subjectivity of weighting procedures and the sensitivity of results to methodological choices. These challenges highlight the need for more robust and objective approaches that can enhance the reliability and interpretability of cross-country health system rankings.

The existing literature on health system performance can broadly be grouped into two main streams. The first stream focuses on the relationship between macroeconomic development and key health indicators, primarily employing regression-based and correlational analyses. The second stream adopts comparative performance frameworks that evaluate countries using multiple indicators, yet often rely on single composite indices or subjective weighting approaches. While these studies provide valuable insights into determinants of health outcomes, relatively few integrate objective weighting with multiple ranking techniques within a unified analytical structure. This gap highlights the need for more robust and methodologically transparent comparative frameworks. Recent studies emphasize the increasing use of composite indices and multi-criteria frameworks in cross-country health system evaluations, highlighting both their analytical advantages and methodological challenges [14]. In particular, concerns regarding subjectivity in weighting procedures and sensitivity to methodological choices have been frequently discussed in the literature, reinforcing the need for integrated and objective evaluation approaches.

In addition, several empirical studies have evaluated the efficiency of health systems across OECD countries using quantitative methods, highlighting significant variations in performance and resource utilization [15]. Despite the growing body of research on cross-country health performance, methodological heterogeneity remains evident across studies. Table 1 summarizes the overall landscape of the literature by presenting together studies that examine health performance within the framework of socioeconomic determinants and those that analyze countries’ health systems using a comparative performance assessment approach.

In the existing literature, a large proportion of studies examining health system performance focus either on single health outcomes such as life expectancy or infant mortality or conduct cross-country comparisons based on a limited number of indicators. While these studies make important contributions to understanding the multidimensional nature of health performance, they remain limited in terms of evaluating countries’ overall health system performance within a holistic and comparable framework.

The literature summarized in Table 1 indicates that studies on health performance are largely structured around two main analytical axes. The first group of studies focuses on examining causal relationships between health outcomes and socioeconomic and structural determinants, whereas the second group approaches countries’ health systems from a comparative performance assessment perspective using multiple indicators. Table 1 summarizes thirteen selected studies examining countries’ health system performance between 1975 and 2025, presenting together studies that analyze health performance within the framework of socioeconomic determinants and those that evaluate countries’ health systems using comparative performance assessment approaches.

Early studies adopted the relationship between economic development and health outcomes as a fundamental analytical framework and demonstrated strong associations between income level and indicators such as life expectancy and mortality [5]. However, over time, the observation of substantial differences in health performance even among countries with similar income levels has shown that health outcomes cannot be explained solely by economic factors. In this context, the decisive role of structural factors such as political–economic structure, public health policies, education level, and income distribution on health performance has been increasingly emphasized in the literature [6,7,13].

More recent studies presented in Table 1 have comparatively evaluated countries’ health systems by considering a wide range of indicators and have demonstrated that the relationship between health expenditure and health outcomes is not linear, and that significant performance differences exist between countries even at similar levels of spending [8,9]. These findings clearly indicate that evaluating health system performance based on a single indicator or simple ratios is insufficient.

In a substantial proportion of existing MCDM-based studies, criterion weights are determined equally or based on subjective assessments. This raises concerns regarding the method sensitivity and comparability of the resulting rankings. In addition, the use of a single ranking method in most studies makes it difficult to assess the extent to which results depend on methodological choices.

Building on the methodological limitations identified in the literature summarized in Table 1, this study evaluates the health system performance of OECD countries using an integrated MCDM framework based on objective weighting. Accordingly, criterion weights are determined objectively using the CRITIC method, and country performance rankings are obtained through the combined use of the MAIRCA and MARCOS methods. Examining the results derived from different ranking methods together allows for an assessment of the sensitivity of countries’ relative performance to methodological choices.

In this respect, the study provides a methodologically consistent and application-oriented contribution to the literature by enabling a multidimensional and comparable evaluation of health system performance. Accordingly, this study contributes to the existing literature by proposing an integrated objective MCDM framework that addresses these methodological limitations and provides a more robust basis for comparative health system ranking and supports evidence-based policy making.

## 3. Materials and Methods

### 3.1. Data Set

In this study, all indicators were obtained exclusively from OECD reports and databases [3,4] in order to ensure data consistency and cross-country comparability. As international organizations such as the World Bank and the OECD may report similar health indicators using different definitions, scopes, and calculation methods, the combined use of multiple data sources may lead to methodological inconsistencies. Therefore, a single data source was preferred.

Differences were observed in the reference years of certain indicators reported in the OECD Health at a Glance reports for 2024 and 2025 [3,4]. To avoid temporal bias in cross-country comparisons, 2021—the most recent year for which all selected indicators were jointly available—was chosen as the reference year. Countries with incomplete observations for this year were excluded from the analysis rather than applying imputation techniques, in order to prevent potential estimation bias in the ranking results. Consequently, the final dataset consists of 27 OECD countries with complete and comparable data for six selected indicators.

The dimensions, codes, and optimization directions of the indicators used in the analysis are presented in Table 2.

Table 2 presents the dimensions, codes, optimization directions, and definitions of the indicators used in the analysis. The selected indicators reflect the multidimensional nature of health system performance by jointly considering health outcomes (life expectancy, avoidable mortality, infant mortality rate, and maternal mortality) and system related inputs or burdens (health spending per capita and chronic disease morbidity). Indicators that contribute positively to performance are modelled as benefit oriented (Max), while those representing adverse health outcomes are modelled as cost oriented (Min). This classification serves to ensure methodological consistency in the subsequent weighting and ranking stages of the analysis. Accordingly, benefit criteria were maximized and cost criteria were minimized during the normalization and ranking procedures.

The selection of these six indicators was guided by two main considerations. First, all variables are consistently reported in OECD Health at a Glance publications using comparable definitions across countries, which ensures cross-country consistency. Second, the chosen indicators jointly reflect both outcome-based measures (such as life expectancy and mortality indicators) and system-related input or burden measures (such as health spending per capita and chronic disease morbidity). Although other dimensions of health systems—such as accessibility, quality of care, and equity—are also important, comparable and complete data for these dimensions were not simultaneously available for all OECD countries for the selected reference year. Therefore, the analysis focuses on a balanced and data-consistent subset of indicators that capture core aspects of health system effectiveness and efficiency.

### 3.2. Methods

The methodological framework of the study consists of two main components: objective weighting and multi-criteria ranking. First, the weights of the six selected indicators were calculated using the CRITIC method [23]. Subsequently, the MAIRCA [24] and MARCOS [25] methods were applied to rank OECD countries based on the derived weights. The selection of the CRITIC method is particularly motivated by its ability to derive objective weights based on the informational content of indicators, thereby reducing subjectivity in multi-dimensional health system evaluation. Given the heterogeneity of health indicators across OECD countries, this approach enables a more data-driven representation of criterion importance.

Similarly, the combined use of MAIRCA and MARCOS is intended to provide complementary ranking perspectives rather than relying on a single methodological structure. This integration allows for cross-validation of results and enhances the interpretability and robustness of the findings within a comparative policy-oriented framework.

The CRITIC method, originally proposed by Diakoulaki et al. [23], determines criterion weights objectively by considering both the contrast intensity (standard deviation) and the degree of conflict (correlation) among indicators. In the context of health system performance, where indicators may exhibit variability and inter-correlation (e.g., expenditure and outcome measures), CRITIC reduces subjectivity by assigning higher weights to indicators with greater informational content and lower redundancy. This feature makes it particularly suitable for multi-dimensional health performance evaluation, as it captures both dispersion and inter-criteria relationships within the dataset.

For the ranking stage, two complementary MCDM methods—MAIRCA [24] and MARCOS [25]—were employed. MAIRCA evaluates alternatives by comparing theoretical and empirical assessment matrices, enabling a balanced comparison of country performances. MARCOS, on the other hand, determines the utility degree of each alternative relative to ideal and anti-ideal solutions. The joint application of these two ranking methods allows for cross-verification of results and strengthens the robustness of the findings by mitigating potential method-dependent bias. This combined use also addresses the limitations of relying on a single ranking technique, as highlighted in the literature.

**Figure 1:**
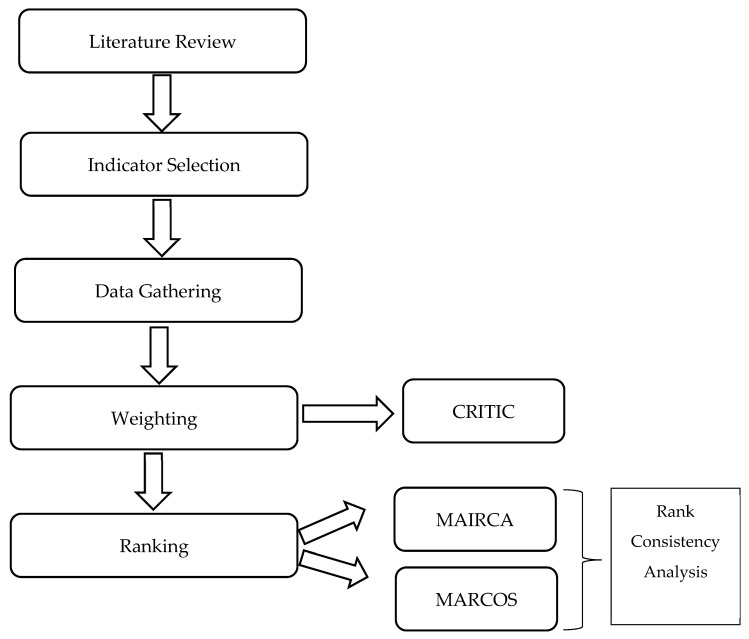
The research methodology.

The methodology employed in the study consists of the following stages (Figure 1).

As shown in Figure 1, the overall analytical framework of the study consists of sequential weighting and ranking stages. The selection of the CRITIC method for criterion weighting is particularly suitable in the context of health system performance assessment, as health indicators often exhibit substantial intercorrelations and heterogeneous dispersion structures. By simultaneously accounting for both the contrast intensity of each criterion and its correlation with other indicators, CRITIC enables the extraction of information-based weights without relying on subjective judgments. Furthermore, the combined application of MAIRCA and MARCOS allows the evaluation of country performance from complementary analytical perspectives. While MAIRCA focuses on deviations between ideal and realized performance levels, MARCOS evaluates alternatives relative to both ideal and anti-ideal reference points. The joint use of these methods therefore enhances result robustness by reducing dependence on a single ranking logic.

#### 3.2.1. CRITIC Method

The CRITIC (Criteria Importance Through Intercriteria Correlation) method is a technique developed to determine the objective weights of criteria in multi-criteria decision-making problems. When determining the importance levels of criteria, the method takes into account both the standard deviations of the criteria and the correlations between them [23]. The steps of the CRITIC method are as follows [23].

Step 1: Construction of the decision matrix

The decision matrix contains the criterion values corresponding to different alternatives. The decision matrix *X* is presented in Equation (1).

(1) $$X={\left[x_{ij}\right]}_{mxn}=\left[\begin{array}{ccc} x_{11} & \ldots & x_{1n}\\ \vdots & \ddots & \vdots \\ x_{m1} & \ldots & x_{mn} \end{array}\right]\;i=1,2,\ldots ,m\;j=1,2,\ldots ,n$$

Step 2: Construction of the normalized decision matrix

The decision matrix is normalized depending on whether the criteria are maximization or minimization oriented.

(2) $$r_{ij}=\frac{x_{ij}-x_{j}^{min}}{x_{j}^{max}-x_{j}^{min\;}}\;max\;oriented$$

(3) $$r_{ij}=\frac{x_{j}^{max}-x_{ij}}{x_{j}^{max}-x_{j}^{min\;}}\;min\;oriented$$

Step 3: Construction of the correlation matrix

Using the data obtained from the normalized decision matrix, the correlation matrix is constructed (Equation (4)).

(4) $$\rho_{jk}=\frac{\sum_{i=1}^{m}\left(r_{ij}-{\bar{r}}_{j})(r_{ik}-{\bar{r}}_{k}\right)}{\sqrt{\sum_{i=1}^{m}{\left(r_{ij}-{\bar{r}}_{j}\right)}^{2}\sum_{i=1}^{m}{\left(r_{ik}-{\bar{r}}_{k}\right)}^{2}}}\;\;j,k=1,2,\ldots ,n$$

Step 4: Calculation of C_j_ value

The C_j_ value is calculated using the standard deviations of the columns in the normalized decision matrix. Equation (5) is used to calculate $\sigma_{j}$, and Equation (6) is used to calculate C_j_.

(5) $$\sigma_{j}=\sqrt{\frac{\sum_{i=1}^{m}{\left(r_{ij}-{\bar{r}}_{j}\right)}^{2}}{m-1}}$$

(6) $$C_{j}=\sigma_{j}\sum_{k=1}^{n}\left(1-\rho_{jk}\right)\;j=1,2,\ldots n$$

Step 5: Calculation of criterion weights

The objective weights of the criteria are calculated (Equation (7)).

(7) $$W_{j}=\frac{C_{j}}{\sum_{k=1}^{n}C_{k}}\;\;\;\;j,k=1,2,\ldots ,n$$

#### 3.2.2. MAIRCA Method

The MAIRCA (Multi Attributive Ideal Real Comparative Analysis) method evaluates alternatives based on the differences between ideal and real performance values. It allows decision makers to directly measure how close alternatives are to ideal targets, thereby producing more balanced and objective ranking results [26]. The steps of the MAIRCA method are as follows [26].

Step 1: Construction of the decision matrix

(8) $$X={\left[x_{ij}\right]}_{mxn}=\left[\begin{array}{ccc} x_{11} & \ldots & x_{1n}\\ \vdots & \ddots & \vdots \\ x_{m1} & \ldots & x_{mn} \end{array}\right]\;i=1,2,\ldots ,m\;j=1,2,\ldots ,n$$

Step 2: Determination of preference priorities of alternatives

(9) $${PA}_{i}=\frac{1}{m},\;\sum_{i=1}^{m}{PA}_{i}=1$$

Step 3: Construction of the ideal expectation matrix

(10) $$T_{pij}={PA}_{i}w_{j}$$

Step 4: Construction of the real evaluation matrix

(11) $$T_{rij}=T_{pij}\frac{x_{ij}-{min}_{k}x_{kj}}{{max}_{k}x_{kj}-{min}_{k}x_{kj}},\;max\;oriented$$

(12) $$T_{rij}=T_{pij}\frac{{max}_{k}x_{kj}-x_{ij}}{{max}_{k}x_{kj}-{min}_{k}x_{kj}},\;min\;oriented$$

Step 5: Construction of the gap matrix

(13) $$g_{pij}=T_{pij}-T_{rij}$$

Step 6: Calculation of the total gap value

(14) $$G_{i}=\sum_{j=1}^{n}g_{ij}$$

Step 7: Ranking of alternatives

The alternative with the lowest total gap score is the closest to the ideal performance. This alternative is the most preferred.

#### 3.2.3. MARCOS Method

The MARCOS (Measurement of Alternatives and Ranking according to Compromise Solution) method was first introduced by Stević et al. [25]. This method is a multi-criteria decision-making (MCDM) technique based on evaluating and ranking alternatives relative to ideal and anti-ideal (reference) solutions [25]. The method offers stable and consistent results when considering a large number of criteria and alternatives, demonstrates high reliability and ranking stability across different scale types, and enables the definition of a new utility function linked to reference points, providing clearer and more interpretable outputs for decision makers [25]. The steps of the MARCOS method are as follows [25].

Step 1: Construction of the decision matrix

(15) $$X={\left[x_{ij}\right]}_{mxn}=\left[\begin{array}{ccc} x_{11} & \ldots & x_{1n}\\ \vdots & \ddots & \vdots \\ x_{m1} & \ldots & x_{mn} \end{array}\right]\;i=1,2,\ldots ,m\;j=1,2,\ldots ,n$$

(16) $$x_{AI,j}={max}_{i}x_{ij\;},\;max\;oriented$$

(17) $$x_{AAI,j}={min}_{i}x_{ij\;},\;max\;oriented$$

(18) $$x_{AI,j}={min}_{i}x_{ij\;},\;min\;oriented$$

(19) $$x_{AAI,j}={max}_{i}x_{ij\;},\;min\;oriented$$

The $x_{AI,j}$ and ${\;x}_{AAI,j}$ solutions are added to the X matrix.

Step 2: Construction of the normalized decision matrix

(20) $$n_{ij}=\frac{x_{ij}}{x_{AI,j}}\;max\;oriented$$

(21) $$n_{ij}=\frac{x_{AAI,j}}{x_{ij}}\;min\;oriented$$

Step 3: Construction of the weighted normalized matrix

(22) $$v_{ij}=n_{ij}w_{j}$$

Step 4: Calculation of utility functions of alternatives

(23) $$S_{i}=\sum_{j=1}^{n}v_{ij}$$

(24) $$S_{AI}=\sum_{j=1}^{n}v_{AI,j}$$

(25) $$S_{AAI}=\sum_{j=1}^{n}v_{AAI,j}$$

Step 5: Calculation of utility degrees

(26) $$K_{i}^{AI}=\frac{S_{i}}{S_{AI}}$$

(27) $$K_{i}^{AAI}=\frac{S_{i}}{S_{AAI}}$$

Step 6: Calculation of the final utility function

(28) $$f_{i}=\frac{1}{2}\left(K_{i}^{AI}+K_{i}^{AAI}\right)$$

Step 7: Ranking of alternatives

${maxf}_{i}$ is the best alternative.

## 4. Results

In the first stage of the analysis process, a decision matrix consisting of health indicators for OECD countries was constructed (Table 3).

Table 3 presents the initial decision matrix including the raw values of the six health indicators for the 27 OECD countries. As shown, substantial cross-country variation is observed across all criteria, particularly in health spending per capita, avoidable mortality, and maternal mortality. Countries such as Switzerland and the United States exhibit high levels of health expenditure, while Mexico and Türkiye display relatively higher mortality-related indicators. These differences provide the empirical basis for the subsequent normalization, weighting, and ranking stages of the analysis.

In the study, the criteria were weighted using the CRITIC method (Table 4). The criteria were ranked in the order C2 > C1 > C3 > C5 > C6 > C4. Health spending per capita emerged as the indicator with the highest level of importance. The second most important indicator was life expectancy. Chronic disease morbidity was identified as the least important indicator.

As reported in Table 4, the weights obtained using the CRITIC method were used as inputs in the analyses with the MAIRCA and MARCOS methods and applied in the weighting of the normalized matrices. In the study, country rankings were obtained using the MAIRCA and MARCOS methods. The results obtained using MAIRCA and MARCOS are presented in Table 5.

As shown in Table 5, the rankings obtained from the MAIRCA and MARCOS methods, based on the CRITIC-derived weights, reveal clear performance differences among OECD countries while exhibiting a largely consistent ranking structure. Switzerland ranks first under both methods, followed by countries such as Sweden, Denmark, Japan, and Israel, indicating stable and high health system performance across alternative ranking approaches. These findings provide important insights for policymakers by highlighting the structural differences in health system performance across OECD countries.

Countries including Germany, Austria, the Netherlands, Spain, and Italy occupy middle positions under both methods, with minor rank shifts attributable to differences in computational logic. In contrast, Mexico, Türkiye, Latvia, Costa Rica, and Chile appear in the lower part of the rankings in both models, with Mexico ranking last in both cases.

To formally assess ranking stability, Spearman’s rank correlation coefficient was calculated between the MAIRCA and MARCOS results. The coefficient was found to be ρ = 0.959, indicating a very strong positive association between the two ranking approaches. This high correlation confirms that country positions remain highly stable across methods despite differences in algorithmic structure.

Overall, the strong concordance between ranking results strengthens the methodological robustness of the integrated MCDM framework and supports the reliability of the obtained health system performance evaluations.

## 5. Discussion

In this section, the empirical findings obtained are discussed in the context of previous studies that examine the health system performance of OECD countries within a multi-dimensional framework and the multi-criteria decision-making approaches employed. The findings of this study show that when health system performance among OECD countries is evaluated using multi-dimensional indicators, substantial differences emerge. The combined use of objective weights obtained through the CRITIC method with the MAIRCA and MARCOS methods indicates that country rankings are largely consistent and supports the methodological reliability of the findings. It is observed that countries ranking at the top (e.g., Switzerland, Sweden, Denmark, Japan) possess not only higher life expectancy and lower avoidable mortality levels, but also a more balanced performance structure associated with resource allocation and system efficiency. In contrast, the observation of small differences between methods in the middle group suggests that ranking results may be partially influenced by method sensitivity [8,9]. A closer inspection of the ranking results shows that minor position shifts mainly occur among middle-performing countries such as Austria, Italy, the Netherlands, and Spain. These differences do not alter the overall performance grouping but reflect the sensitivity of ranking methods to relative distances between closely performing countries. In other words, when countries display similar performance profiles across multiple indicators, small variations in weighting or distance calculation mechanisms may lead to modest changes in ordinal positions. However, such shifts remain limited in magnitude and do not affect the stability of the top and bottom performance groups. This indicates that, particularly among countries with similar performance levels, the weighting and ranking techniques used can play a more decisive role in shaping results. This finding is consistent with previous studies emphasizing that evaluating health system performance using a single indicator or a single method may be limited [6,7]. These studies also note that the relationship between health expenditure and health outcomes is neither linear nor one-dimensional, and therefore multi-dimensional evaluation approaches provide more realistic results. The combined application of CRITIC based objective weighting with the MAIRCA and MARCOS methods in this study provides methodological support for these findings in the literature. Overall, rather than relying on a single indicator or a single method, the joint use of objective weighting and multiple ranking approaches can generate more balanced and reliable results. These findings directly address the stated sub-aims of the study by demonstrating (i) the effectiveness of objective weighting through CRITIC, (ii) the comparative ranking of OECD countries using two complementary MCDM approaches, and (iii) the robustness of results through high rank consistency between methods. In addition, the ranking patterns obtained in this study are broadly aligned with widely recognized international health system performance assessments (e.g., OECD and WHO-based evaluations), where countries such as Switzerland, Sweden, and Denmark consistently appear among the top performers. This consistency provides an external validation of the proposed framework and strengthens the credibility of the results.

It should also be noted that OECD countries differ substantially in terms of health system organization, financing mechanisms, and ownership structures, ranging from predominantly publicly funded systems to mixed or more market-oriented models. Although the present analysis does not explicitly categorize countries based on ownership types, variations in institutional design may partially explain observed performance differences. The findings therefore reflect measurable outcome and efficiency dimensions rather than structural or governance-specific characteristics. Future research could integrate institutional typologies or ownership variables to further explore how organizational models influence comparative health system performance.

From a policy making perspective, this finding highlights that decisions aimed at improving health system performance should not be based on a single indicator or method and underscores the importance of holistic evaluation tools. For policy makers, these results indicate that improving health system performance is possible not only through increasing health expenditure, but also through more efficient and targeted allocation of resources. More specifically, for lower-ranked countries such as Mexico and Türkiye, improvements in preventable and maternal mortality, as well as enhanced primary healthcare coverage, appear to be particularly critical. The findings suggest that merely increasing total health expenditure may not be sufficient unless accompanied by institutional reforms that improve efficiency, access, and preventive care services. Therefore, policy efforts should focus not only on resource expansion but also on strengthening system organization and targeted health interventions.

The study is based on a limited set of six indicators that are consistently available across OECD countries for the selected reference year. While this approach ensures cross-country data comparability and methodological consistency, it inevitably narrows the scope of performance assessment. Health systems are inherently multi-dimensional and may differ in the range of indicators emphasized, including accessibility, quality of care, preventive services, and social care integration. The selected indicators primarily reflect outcome- and expenditure-based dimensions in line with OECD data availability constraints, which may influence the relative positioning of countries. Therefore, the findings should be interpreted within the boundaries of the selected indicator framework. In addition, the ranking results presented in this study may serve as a practical benchmarking tool for policy makers by providing a structured comparison of country performance across multiple health indicators. Rather than offering a single definitive assessment, the framework enables the identification of relative strengths and weaknesses across countries and may support more informed policy evaluation and resource allocation decisions.

## 6. Conclusions

This study evaluated the health system performance of OECD countries within an integrated multi-criteria decision-making (MCDM) framework based on six indicators and generated country rankings using the MAIRCA and MARCOS methods with objective weights determined by the CRITIC method. The findings indicate that there are pronounced differences in performance among countries and that countries in the top group consistently stand out under both methods. The fact that differences between methods emerge mainly at a limited level within the middle group suggests that the rankings are generally reliable. Overall, the combined use of objective weighting and multiple ranking methods provides a useful analytical framework for evaluating health system performance in a more balanced and evidence-based manner.

The results reveal that there are significant differences in health system performance among OECD countries. Countries such as Switzerland, Sweden, Denmark, Japan, and Israel rank at the top under both methods, demonstrating high and stable performance. In contrast, countries such as Mexico, Türkiye, Latvia, and Costa Rica are positioned at the lower end of the rankings. These results indicate that indicators such as health spending per capita, life expectancy, avoidable mortality, and maternal and infant mortality rates play a decisive role in determining health system performance.

The high level of consistency between the rankings obtained from the MAIRCA and MARCOS methods supports the reliability of the integrated MCDM approach used in this study. The limited differences observed between methods are mainly evident among countries in the middle performance group. This situation arises from differences in the sensitivity of various MCDM methods to the criteria. This finding demonstrates that health system performance evaluation should not rely on a single method and that the use of multiple methods yields more robust results.

The study makes a complementary methodological contribution to the literature by jointly applying CRITIC based objective weighting with the MAIRCA and MARCOS methods in the evaluation of health system performance. In this respect, the study shows that the combined use of CRITIC based objective weighting and the MAIRCA and MARCOS methods provides a complementary and applicable analytical framework in the literature. The results offer an analytical framework for policy makers to identify countries’ strengths and areas requiring improvement and contribute to the development of evidence-based health policies.

### Limitations and Future Directions

This study has several limitations. First, the analysis is limited to 27 countries with complete data available in OECD reports, which restricts the generalizability of the results to non-OECD countries. Second, although the indicators used in the analysis cover key variables representing health system performance, dimensions such as the quality of healthcare services, accessibility, and regional inequalities could not be included due to data constraints.

In addition, the study is based on cross-sectional data and does not capture changes over time. Analyses conducted using data from different years or employing panel data could reveal the dynamic nature of health system performance in greater detail.

In future studies, the combined use of different objective and subjective weighting methods may allow for comparisons of results in terms of method sensitivity. Moreover, the use of panel data structures would enable the analysis of temporal changes in health system performance. Incorporating indicators that reflect the quality dimension of healthcare services and measures of inequality into the analysis would contribute to more comprehensive policy implications. In this context, comparative analyses that jointly consider different data sources and alternative weighting approaches may contribute to a deeper understanding of health system performance.

## Figures and Tables

**Table 1:** Summary of selected studies examining countries’ health performance.

Authors (Year)	Objective	Method	Findings/Results
Preston, 1975 [5]	To examine changes in the relationship between economic development and mortality in the 20th century.	Cross sectional country analysis, correlations, regression curves, comparative analysis.	- A strong correlation was found between income level and life expectancy (r ≈ 0.8). - Over time, higher life expectancy is observed at the same income level. - The effect of income growth is more pronounced in low-income countries.
Cereseto & Waitzkin, 1986 [6]	To analyse the relationship between economic development, political economic systems, and quality of life.	Statistical regression and correlation analyses; comparison of capitalist and socialist country groups.	Socialist countries exhibit better health indicators compared to capitalist countries with similar GNI levels. Political economic structure is a key determinant of health performance.
Belek & Belek, 2002 [16]	To identify socioeconomic factors affecting countries’ health performance.	UNICEF, World Bank, and WHO data (1995–1999). Estimation of expected infant mortality via simple regression, comparison with actual values to derive performance scores, followed by multiple regression analysis.	Infant mortality rate is a core health indicator. Key determinants of health performance include the Gini coefficient, primary education enrolment among women, and socialist political structure. Per capita income and health expenditure alone are not decisive. Socialist countries demonstrate higher health performance.
Cutler, Deaton & Lleras-Muney, 2006 [7]	To analyse historical, cross country, and socioeconomic determinants of mortality.	Comprehensive literature review combined with empirical data analysis using examples from the United States, Europe, and developing countries.	Mortality decline occurred in three phases: (i) nutrition in the 18th–19th centuries, (ii) Public health in the 19th–20th centuries, (iii) Medical technology in the 20th century. - The relationship between income and health is not sufficient on its own to explain health outcomes. - Health inequalities are pronounced in low-income countries, with female education being a particularly critical factor.
Joumard, André & Nicq, 2010 [8]	To comparatively evaluate the performance and efficiency of health systems in OECD countries based on health outcomes and institutional indicators.	Comparative analysis of OECD countries using panel data regressions and Data Envelopment Analysis (DEA).	- A strong but non-linear relationship exists between health expenditure and health outcomes across OECD countries. - Significant performance differences exist at similar expenditure levels. - Health system efficiency depends not only on spending but also on institutional structure and policy mix.
Tchouaket, Lamarche, Goulet & Contandriopoulos, 2012 [9]	To evaluate OECD health system performance within a multidimensional framework and classify countries according to similar performance profiles.	Health system indicators from 27 OECD countries analysed using multivariate statistical techniques and cluster analysis.	- Significant heterogeneity in health system performance across OECD countries. - High performing countries generally show better health outcomes and more efficient service delivery. - Health expenditure alone is insufficient to explain high performance.
Pickett & Wilkinson, 2015 [17]	To assess whether the relationship between income inequality and health is causal.	Literature review conducted within the framework of epidemiological causality criteria.	- Numerous studies indicate that higher income inequality is associated with worse health and social outcomes. - The few contradictory findings are largely attributable to methodological limitations.
Yiğit, 2019 [18]	To compare health system performance of OECD countries and determine Türkiye’s position among them.	The use of the TOPSIS method, one of the multi criteria decision making (MCDM) approaches, based on data from 35 OECD countries.	Slovenia, Korea, and Israel exhibit the highest health performance. USA, The United States, Mexico, and Türkiye rank lowest. High health expenditure does not necessarily imply better health outcomes.
Xi, 2020 [19]	To analyse the impact of income inequality (Gini) and GDP per capita on quality of life indicators.	World Bank and UNODC data (post 2010) covering 216 countries. Regression analyses.	- Higher GDP per capita and lower Gini coefficients → higher life expectancy and lower infant mortality. - Infant mortality increases as Gini rises. - Homicide rates are positively associated with Gini but not with GDP per capita.
Uslu, 2021 [20]	To compare the health system performance of OECD countries using multi-criteria decision-making methods and to rank countries according to their performance.	The performance of 26 OECD countries based on 2019 data, using the TOPSIS and VIKOR multi criteria decision making methods.	Rankings obtained from TOPSIS and VIKOR are largely similar and consistent. While the United States and South Korea rank among the top countries under both methods, Mexico ranks last and Türkiye ranks 25th among the 26 OECD countries.
Acar, 2022 [21]	To compare OECD countries’ performance within the framework of global socio-economic indices using a multi-criteria decision-making approach.	CRITIC method applied to OECD data for the 2015–2019 period.	Denmark ranks highest and Mexico lowest among OECD countries. The Gini index consistently receives the highest weight using CRITIC, indicating income inequality as a key determinant of overall performance.
Li, 2024 [22]	To examine the relationship between GDP per capita and infant mortality rate across 217 countries during 2000–2022.	Panel data analysis (4995 observations) using World Bank data. Multiple regression, interaction models, log and quadratic models.	- Infant mortality declines as GDP per capita increases. - Education expenditure, health expenditure, clean energy access, and electricity access reduce infant mortality. - Female unemployment has a weak positive effect. - Health expenditure alone is not statistically significant. - Interaction terms (GDP × education/health expenditure) are significant.
Yılmaz (2025) [10]	To evaluate the health system performance of OECD countries using a multi-criteria decision-making framework.	MULTIMOORA method	The results indicate notable differences in health system performance among OECD countries and highlight the usefulness of MCDM techniques for cross-country health performance evaluation.

**Table 2:** Dimensions, codes, directions and indicators used in the analysis.

Dimensions	Codes	Directions	Indicators
Life expectancy	C1	Benefit (Max)	The average number of years a newborn is expected to live, assuming that current mortality rates remain constant throughout their lifetime in a given country.
Health spending per capita	C2	Benefit (Max)	The total annual health expenditure per capita in a country, including both public and private sources.
Avoidable mortality	C3	Cost (Min)	The mortality rate attributable to causes that could be prevented or treated through appropriate healthcare services and effective preventive interventions.
Chronic disease morbidity	C4	Cost (Min)	The disease burden attributable to chronic diseases or the prevalence of these diseases within the population.
Infant mortality rate	C5	Cost (Min)	The annual number of deaths of infants who die before reaching one year of age per 1000 live births in a given country.
Maternal mortality	C6	Cost (Min)	The frequency of maternal deaths resulting from causes related to pregnancy, childbirth, or the postpartum period within a given population.

**Table 3:** Decision matrix.

Country	Criteria					
	Life Expectancy	Health Spending per Capita	Avoidable Mortality	Chronic Disease Morbidity	Infant Mortality Rate	Maternal Mortality
Australia	83.2	6730.995117	156	8.1	3.2	4.7
Austria	81.4	5851.962402	187	5.2	2.4	3.6
Belgium	81.8	5404.624023	184	6.7	2.9	8.8
Canada	81.3	6255.027832	184	6.8	4.7	8.5
Chile	81.2	1547.289673	229	14	5.9	11.6
Costa Rica	80.9	978.8415527	241	23.2	9.5	15
Czechia	79	2431.084961	250	7.7	2.3	4
Denmark	81.3	6456.083984	175	2.3	2.3	3.4
Germany	80.7	6182.34375	195	6.6	3.2	4.1
Greece	80.8	1768.110718	213	7.2	3	3.9
Hungary	76	1224.433838	422	11.2	3.6	7.9
Israel	82.8	4223.872559	134	7.9	2.9	1.1
Italy	82.8	3134.680664	135	7.2	2.3	3.6
Japan	84.1	3889.357178	131	6.4	1.8	4.3
Korea	82.7	3049.671631	151	10.4	2.3	8.4
Latvia	74.5	1642.471313	449	9.3	2.4	31.6
Mexico	75.2	650.9707031	418	14.3	12.1	38.2
Netherlands	81.7	5796.034668	156	6.4	3.2	3
Poland	77.2	1193.130615	316	10.8	3.8	2
Portugal	81.8	2580.756348	180	7.4	2.6	13.1
Slovak Republic	77	4021	341	8.9	5.4	3.8
Slovenia	81.3	5527	210	10.8	2.5	11.5
Spain	83.2	2910.837646	154	3.6	2.6	3.3
Sweden	83.1	5943.361328	138	5.1	2.2	4.8
Switzerland	83.7	10,963.43066	124	4.3	3.8	1.2
Türkiye	77.3	385.8799744	243	16.6	9.1	12.6
United States	77.5	12,434.43359	312	12.5	5.6	22.3

Source: OECD Health at a Glance reports 2024–2025, OECD Indicators [3,4].

**Table 4:** Weights of criteria.

C1	C2	C3	C4	C5	C6
0.16399	0.22255	0.162676	0.134833	0.160519	0.155432

**Table 5:** MAIRCA and MARCOS method results.

Country	MAIRCA		MARCOS	
	Q_i_	Rank	F (K_i_)	Rank
Australia	0.007816978	5	0.551572645	10
Austria	0.008806488	9	0.572495183	6
Belgium	0.010257712	12	0.507832173	12
Canada	0.011008578	13	0.484977857	16
Chile	0.018020733	20	0.344133010	23
**Costa Rica**	0.023625604	24	0.300322345	25
Czechia	0.014434814	18	0.465455766	17
**Denmark**	0.007452328	4	0.669277009	2
Germany	0.010045510	11	0.526209409	11
Greece	0.013332668	17	0.446566390	19
**Hungary**	0.022538774	23	0.332206408	24
Israel	0.008597807	6	0.651894844	3
Italy	0.009235821	10	0.557500453	9
**Japan**	0.007451768	3	0.608313386	5
Korea	0.011163273	14	0.502109836	13
Latvia	0.026233206	26	0.362068924	22
**Mexico**	0.033711558	27	0.245292106	27
Netherlands	0.008735633	7	0.562207960	8
Poland	0.018940257	22	0.407213407	20
Portugal	0.012776727	16	0.462552104	18
Slovak Republic	0.018344506	21	0.396362923	21
Slovenia	0.012140059	15	0.490098793	15
Spain	0.008754547	8	0.568238873	7
**Sweden**	0.006806885	2	0.61407414	4
**Switzerland**	0.002907196	1	0.776970011	1
**Türkiye**	0.024165697	25	0.291241613	26
United States	0.015581021	19	0.499402269	14

Note: Countries consistently ranked within the top five or bottom five positions under both MAIRCA and MARCOS methods are highlighted in bold to emphasize ranking stability.

## Data Availability

The data used in this study are publicly available from OECD Health at a Glance reports (2024 and 2025).
